# HPLC-UV/HRMS methods for the unambiguous detection of adulterations of *Ginkgo biloba* leaves with *Sophora japonica* fruits on an extract level

**DOI:** 10.1080/13880209.2021.1910717

**Published:** 2021-04-22

**Authors:** Evangelia Bampali, Stefan Germer, Rudolf Bauer, Žarko Kulić

**Affiliations:** a Institute of Pharmaceutical Sciences, Section of Pharmacognosy, University of Graz, Graz, Austria; b Preclinical Research & Development, Dr. Willmar Schwabe GmbH & Co., Karlsruhe, Germany; c Analytical Development, Dr. Willmar Schwabe GmbH & Co., Karlsruhe, Germany

**Keywords:** Genistein, sophoricoside, marker compounds, quality control

## Abstract

**Context:**

*Ginkgo biloba* L. (Ginkgoaceae) leaf extract is one of the most frequently sold herbal extracts. There have been reports on poor quality and adulteration of ginkgo leaf extracts or the powdered plant material with extracts or powder of *Styphnolobium japonicum* (L.) Schott (Fabaceae) (syn. *Sophora japonica* L.) fruits, which is rich in flavone glycosides.

**Objective:**

The study investigates whether ginkgo leaves genuinely contain genistein and sophoricoside and whether these two substances could be used as markers to detect adulterations with sophora fruits.

**Materials and methods:**

A total of 33 samples of dried ginkgo leaves were sourced from controlled plantations in China, the USA, and France. After extraction, the samples were analyzed using two high-performance liquid chromatography (HPLC) coupled with UV/HRMS methods for the detection of genistein and sophoricoside, respectively. Chromatograms were compared to standard reference materials.

**Results:**

In none of the tested ginkgo samples, neither genistein nor sophoricoside could be detected. The applied method was designed to separate genistein from apigenin. The latter is a genuine compound of ginkgo leaves, and its peak may have been previously misidentified as genistein because of the same molecular mass. The method for the detection of sophoricoside allows identification of the adulteration with sophora fruit without prior hydrolysis. By both HPLC methods, it was possible to detect adulterations of ≥2% sophora fruits in the investigated ginkgo extract.

**Conclusion:**

The methods allow unambiguous detection of adulterations of ginkgo leaves with sophora fruits, using genistein and sophoricoside as marker compounds.

## Introduction

*Ginkgo biloba* L. (Ginkgoaceae) is one of the oldest living tree species existing on earth for 200 million years. Extracts prepared from its leaves are among the top-selling herbal products in the world and are sold as active ingredients of numerous dietary supplements, botanicals, herbal medicinal products, and complementary medicines (Liu et al. 2014; Wohlmuth et al. 2014). The majority of ginkgo extracts on the market are made from leaves cultivated at plantations in China, France, and the USA, but there are also cultivations in other agricultural areas located in New Zealand and Korea (Gafner 2018).

Constituents of *G. biloba* comprise many groups of chemical compounds: terpene trilactones (e.g., ginkgolides A, B, C, J, bilobalide), flavonoids (flavanols, flavones, and flavonols based on the aglycones isorhamnetin, kaempferol, quercetin, and myricetin), organic acids (such as shikimic acid, protocatechuic acid, etc.), polyacetate derived compounds, and others, such as carbohydrates, miscellaneous organic compounds, and inorganic ingredients (López-Gutiérrez et al. 2016). The best-studied pharmacologically active compounds in ginkgo leaf extracts are flavonol glycosides and terpene lactones (Liu et al. 2014; Wohlmuth et al. 2014). However, all active constituents of ginkgo extracts are not yet fully defined (Gafner 2018).

*Ginkgo biloba* is one of the most intensely studied medicinal plants. There are numerous studies for pharmacological activities of ginkgo extracts for a range of conditions, including age-associated cognitive decline and dementia, vertigo, tinnitus, and peripheral arterial disease (Horsch and Walther 2004; von Boetticher 2011; Gauthier and Schlaefke 2014; Basta 2017). Based on the traditional medical application, ginkgo leaves are used for the relief of the heaviness of the legs and the sensation of cold hands and feet associated with minor circulatory disorders. Furthermore, a well-established use of specific extracts has been acknowledged by the Committee on Herbal Medicinal Products of the European Medicines Agency for the improvement of (age-associated) cognitive impairment and of quality of life in mild dementia (Committee on Herbal Medicinal Products, European Medicines Agency 2015).

Because of its high economic value, there have been numerous reports of adulteration and poor quality of *G. biloba* leaf extracts (Harnly et al. 2012; Wohlmuth et al. 2014; Booker et al. 2016). Adulteration can be either accidental or intentional and economically motivated, which is mostly done by adding cheaply-sourced flavonol-containing substances (Gafner 2018). The main concern is about the addition of pure flavonols (quercetin, kaempferol, isorhamnetin being the principal aglycones) and flavonol glycosides, or extracts from other botanicals such as *Styphnolobium japonicum* (L.) Schott (Fabaceae) (syn. *Sophora japonica* L.) and *Fagopyrum esculentum* Moench (Polygonaceae), which are rich in flavonol glycosides (Chandra et al. 2011; Wohlmuth et al. 2014; Gafner 2018). Macroscopic identification of adulterations of plant material is not always feasible, since samples may already be in powdered form when purchased from vendors on the global market.

**Figure 1.**
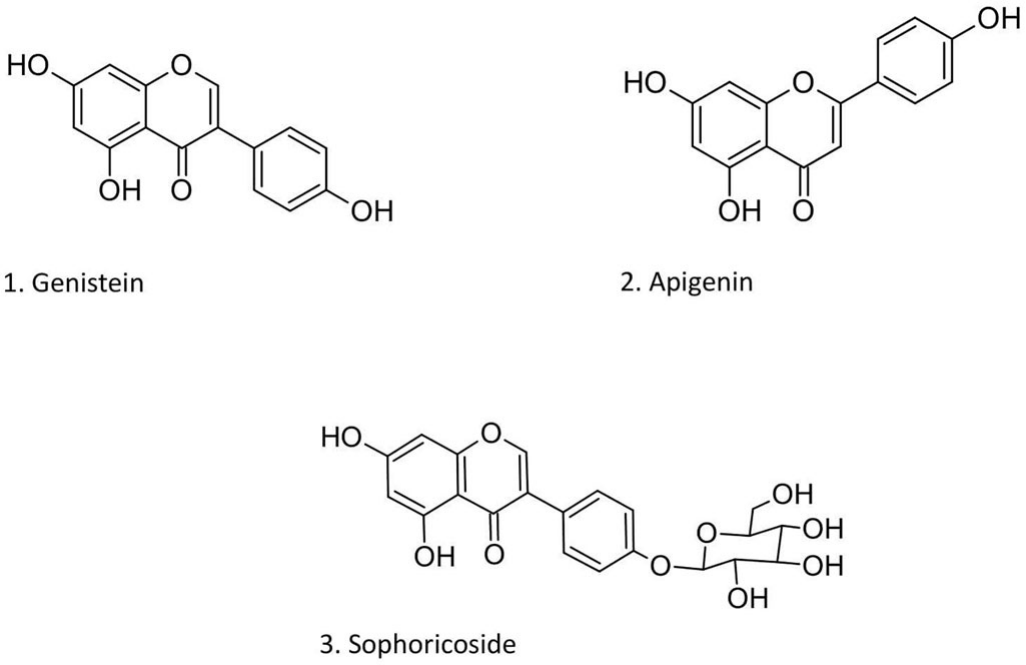
Chemical structures of the isoflavone genistein (**1**), its glucoside sophoricoside (**3**), and the flavone apigenin (**2**).

Sophorae fructus, the dried ripe fruit of *S. japonicum*, is used in Traditional Chinese Medicine (TCM) for its haemostatic properties. Based on the chemical analysis, Sophorae fructus contains flavonoids, alkaloids, terpenoids, amino acids, saccharides, and phospholipids. Isoflavones, such as sophoricoside, genistein, and genistin, and flavonols, like rutin, quercetin, and kaempferol, are the main components (Chang et al. 2012; Chinese Medicine Division, Department of Health 2012) (Figure 1).

It has been suggested that the isoflavone genistein can be used as a marker to detect adulterations of ginkgo leaf extracts with extracts of *S. japonica* (Avula et al. 2015). However, other authors have reported genistein to be a genuine constituent in ginkgo leaf extract, although in very small amounts (Wang et al. 2007). It has also been reported, that the amount of genistein in the leaves was dependent on the season (Yao et al. 2017), or only in leaves, stems, and fruits of male ginkgo trees and not of female trees (Pandey et al. 2014). Moreover, in 2015, a limit of 1% sophoricoside (genistein-4′-*O*-glucoside) in ginkgo extracts was set by the China Food and Drug Administration (China Food and Drug Administration 2015).

Intentional or accidental adulteration of *G. biloba* extract is an ongoing problem. To detect possible adulterations with sophora fruit, the objective of this study was to investigate whether genistein and sophoricoside are genuine constituents of ginkgo leaves. Difficulties in developing a high-performance thin-layer chromatography (HPTLC) method for the detection of genistein in ginkgo leaf extracts (Frommenwiler et al. 2019) along with the wrong assignment of apigenin as genistein because of the same molecular mass, revealed the need for elaborating a more efficient high-performance liquid chromatography (HPLC) method. In this study, HPLC combined with ultraviolet (UV) photodiode array detection and high-resolution mass spectrometry (HRMS) was used to develop methods for unambiguous detection of genistein and sophoricoside in ginkgo leaf and *S. japonica* fruit extracts.

## Materials and methods

### Test samples

Thirty-three dried *G. biloba* leaf samples were obtained from plantations in China (controlled contract cultivation) (12 samples), the USA (South Carolina) (13 samples), and France (Département Gironde) (8 samples), by Dr. Willmar Schwabe GmbH & Co. KG. The herbal drug complies with the requirements described in the monograph of *G. biloba* in the European Pharmacopoeia (European Directorate for the Quality of Medicines and Healthcare, Council of Europe 2019) and this material is used for the production of quantified EGb 761^®^. The various samples were harvested in different years (2013, 2014, 2015, and 2016) from plantations in China, (2013, 2014, 2015, and 2016) from the USA and (2014, 2015, and 2016) from France. Voucher specimens are deposited at Dr. Willmar Schwabe (Karlsruhe, Germany). *Sophora japonica* fruits were obtained from Kräuter Schulte (Gernsbach, Germany) and a voucher specimen is deposited at Dr. Willmar Schwabe (Karlsruhe, Germany).

### Solvents, reagents and chemicals

Ethanol (analytical grade), methanol hypergrade for liquid chromatography (LC)-mass spectrometry (MS), and acetonitrile hypergrade for LC-MS, for extraction and HPLC analysis, were purchased from Merck, Germany. The deionized water was obtained by a water purification system (Evoqua, Water Technologies, Günzburg, Germany). Isopropanol (2-propanol) and hydrochloric acid 32%, for the preparation of the hydrolysis solution, were both purchased from Merck, Germany. For the preparation of the hydrolysis solution, 300 mL isopropanol was added to a 1 L volumetric cylinder and filled up with hydrochloric acid 3 M to the final volume.

### Reference standards

Genistein was purchased from HWI Group (Rülzheim, Germany), sophoricoside from Sigma-Aldrich (St. Louis, USA), and apigenin from Fluka (Buchs, Switzerland).

### Extraction and hydrolysis

Dried *G. biloba* leaves or *S. japonica* fruits were introduced into a flask, and extracted twice (Büchi Rotavapor R-124 and Büchi water bath B-480, both from Büchi, Switzerland) with 60% (v/v) ethanol (1:7, drug to solvent ratio) at 60 °C for 1 h. After cooling at room temperature (25 °C), the two extraction suspensions were filtered (T 1500 filter paper, Pall Corporation, Germany) and mixed. The obtained extract solution was evaporated under vacuum and subsequently lyophilised (Alpha 2–4, Christ, Bühl, Germany). All the produced ginkgo extracts are not commercially available and were only used for these investigations.

In order to test the detectability of adulteration, *G. biloba* leaves were intentionally adulterated with 2% (w/w) *S. japonica* fruits and an extract was prepared according to the procedures described above.

Hydrolysed *G. biloba* and *S. japonica* extract samples were prepared by transferring 20.0 mg of the dry extract into hydrolysis vials and the addition of 1 mL of hydrolysis solution. Subsequently, the solutions were heated at 100 °C in a water bath (Labortechnik HB4 basic, IKA, Staufen im Breisgau, Germany) for 45 min.

### Sample preparation

Reference standards (genistein, sophoricoside, and apigenin) were dissolved in the initial gradient solution of each of the appropriate HPLC methods, at a concentration of 0.5 mg/mL. The samples were sonicated for 10 min at room temperature, then filtered through a filter with pore size 0.45 μm (Rotilabo^®^ PTFE, Carl Roth, Germany) and transferred into individual vials which were subjected to HPLC-UV/HRMS analysis.

For the analysis of genistein, the hydrolyzed mixtures of the 33 ginkgo samples were transferred into individual vials for HPLC-UV/HRMS after filtration through a syringe filter with pore size 0.45 μm (Rotilabo^®^ PTFE, Carl Roth, Germany).

For the analysis of sophoricoside, the 33 samples of lyophilized ginkgo extracts were dissolved at a concentration of 10 mg/mL in the initial gradient solution. The solutions were sonicated for 10 min at room temperature and then transferred into individual vials for HPLC-UV/HRMS analysis after filtration using a 0.45 μm filter (Rotilabo^®^ PTFE, Carl Roth, Germany).

### Apparatus and chromatographic conditions for genistein analysis

HPLC-UV/HRMS was performed using a Thermo Orbitrap Fusion system coupled with a Thermo Vanquish UHPLC using a Waters Cortecs UPLC C18 1.6 M (2.1 × 150 mm) column. The mobile phase consisted of 0.4% aqueous (deionized water) formic acid (phase A) and acetonitrile: methanol (50:50 v/v) LC-MS grade with 0.4% formic acid (phase B). At a flow rate of 0.2 mL/min, the linear gradient was as follows: 0.00–40.00 min, 70–30% (A–B%) to 15–85% (A–B%) followed by a 5 min column wash with 15–85% (A–B%) and 5 min equilibration period with 70–30% (A–B%). UV detection wavelength of 254 nm, a column temperature of 40 °C, and an injection volume of 2 µL were applied.

MS parameters in positive ionization mode were: ionization voltage 3500 V, electrospray ionization (ESI), ion transfer tube temperature 350°, vaporizer temperature 350 °C, 3 scans, resolution of 30000, HCD collision energy (%) 50. System control and data evaluation were performed with Thermo^®^ Xcalibur for LC-MS.

### Apparatus and chromatographic conditions for sophoricoside analysis

The analysis of sophoricoside was performed by a modified HPLC method described in the monograph of Sophorae Fructus in the Hong Kong Chinese Materia Medica Standards (Chinese Medicine Division, Department of Health 2012).

HPLC-UV/HRMS was performed with the same instrument configuration as described above. The mobile phase consisted of 0.4% aqueous (ultra-power water) formic acid (phase A) and acetonitrile: methanol (50:50 v/v) LC-MS grade with 0.4% formic acid (phase B). At a flow rate of 0.2 mL/min, the linear gradient was as follows: 0.00–40.00 min, 85–15% (A–B%) to 70–30% (A–B%) followed by a 5 min column wash with 15–85% (A–B%) and 5 min equilibration period with 85–15% (A–B%). UV detection wavelength of 254 nm, a column temperature of 40 °C, and an injection volume of 2 µL were applied.

MS parameters in the positive ionization mode were: ionization voltage 3500 V, ESI, ion transfer tube temperature 350 °C, vaporizer temperature 350 °C, 3 scans, resolution of 30000, HCD collision energy (%) 50. System control and data evaluation were performed with Thermo^®^ Excalibur for LC-MS.

### Data analysis

ACD/Spectrus Processor (v2017.2.1) software was used to process and analyze all data files.

## Results

### Analysis of genistein

**Figure 2.**
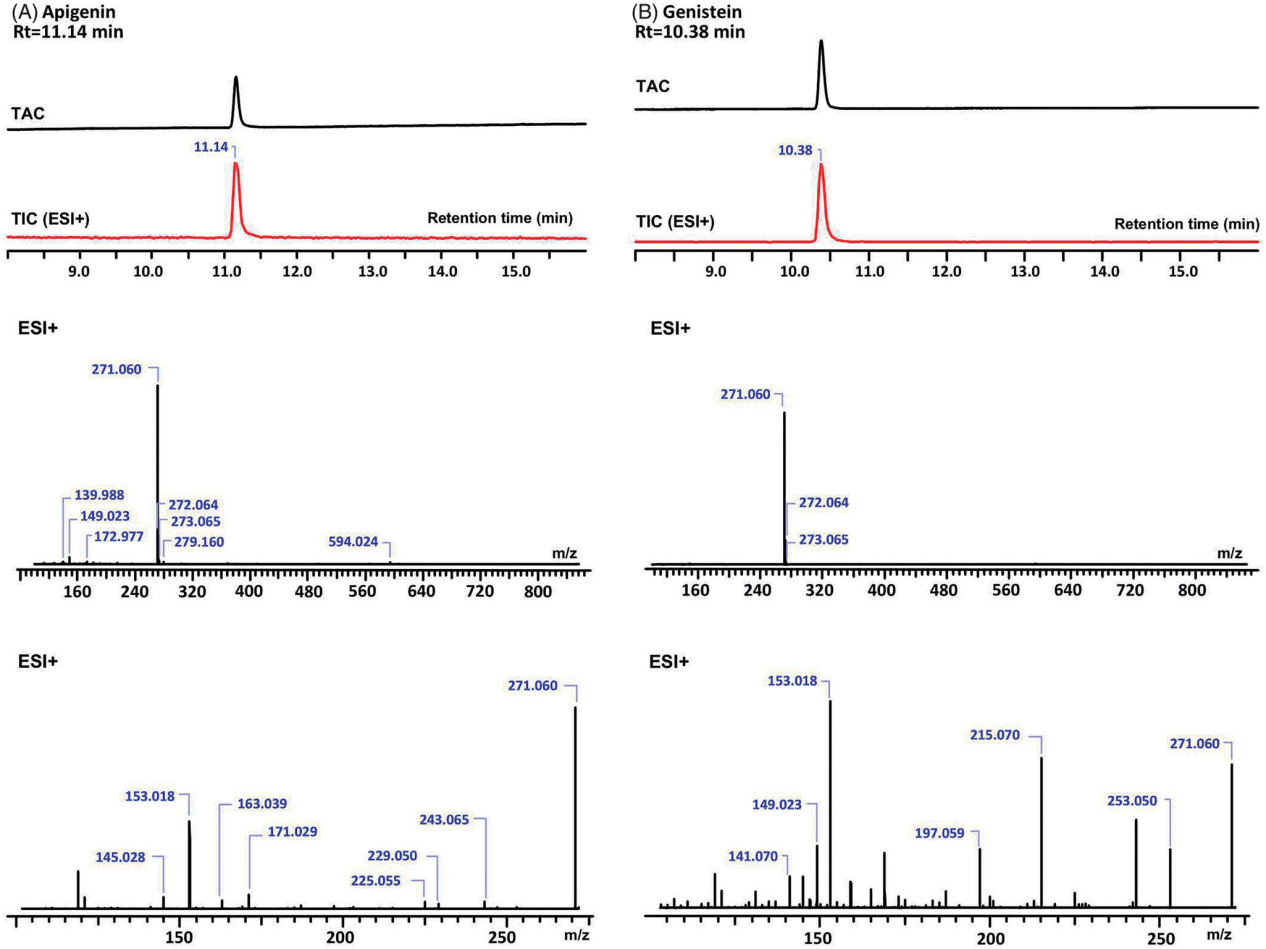
The total absorbance chromatograms (TAC) and total ion chromatograms (TIC) of the reference substances, apigenin (A) and genistein (B) (top) as detected with DAD and their MS (middle) and MS2 (bottom) spectra in the positive ion mode.

A new HPLC-UV/HRMS method was developed to investigate whether genistein is a genuine constituent of *G. biloba* leaf extract. This method features slightly different retention times (*Rt)* of apigenin and genistein (11.14 min and 10.38 min, respectively) (Figure 2). Both compounds have the same molecular mass, which may have led to misinterpretations in studies claiming that genistein is a genuine constituent of *G. biloba* leaves. The new UHPLC method can unambiguously distinguish these two compounds due to different retention times, UV-spectra, and MS fragmentation patterns, and thus, misinterpretations can be avoided (Table 1 and Figure 2).

**Table 1.** Fragmentation pattern of apigenin and genistein.

Compound	Ion mode	Rt (min)	Monoisotopic mass	*m/z*	MS2 fragments +
Apigenin	Positive	11.14	270.0528	271.060	271.060; 243.065; 229.050; 225.055; 171.029; 163.039; 153.018; 145.028
Genistein	Positive	10.38	270.0528	271.060	271.060; 253.050; 215.070; 197.059; 153.018; 149.023; 141.070

**Figure 3.**
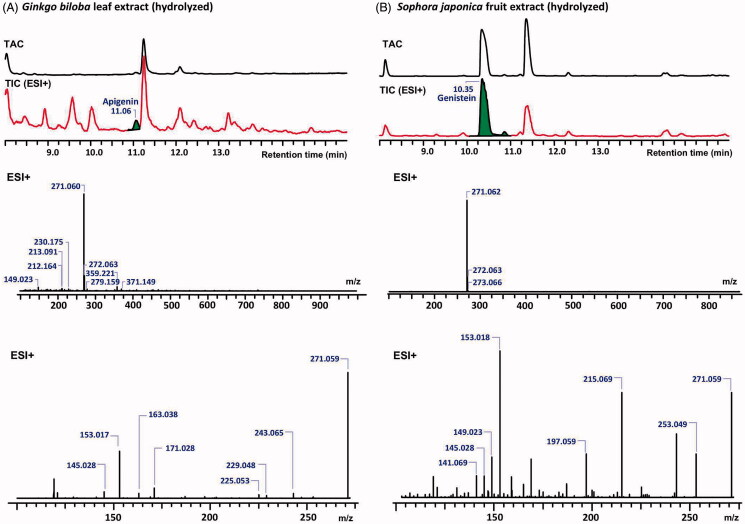
The total absorbance chromatograms (TAC) and total ion chromatograms (TIC) of *G. biloba* leaf extract (A) and *S. japonica* fruit extract (B) (top) as detected with DAD and the identification of apigenin and genistein respectively, with their MS (middle) and MS2 (bottom) spectra in the positive ion mode.

All 33 ginkgo samples described above were analyzed for the presence of genistein by LC-DAD-HRMS using positive selective ion monitoring. The chromatograms of the hydrolyzed *G. biloba* leaf and *S. japonica* fruit extracts are shown in Figure 3. Processing of the UHPLC-DAD-HRMS data of the ginkgo leaf extracts, *S. japonica* fruit extract, and of the reference compounds apigenin and genistein showed that genistein could not be detected in any of the tested ginkgo samples, whereas traces of apigenin were detected instead (Figure 3).

### Analysis of sophoricoside

Besides the method for the specific detection of genistein, one additional method was developed for the detection of sophoricoside, in which hydrolysis of the extract is not necessary. Due to the reduced number of manual steps, this method is more suitable for the screening of a high number of samples. Since sophoricoside is among the major constituents of *S. japonica* fruits (He et al. 2016), adulteration of *G. biloba* leaves with *S. japonica* fruits can be detected using sophoricoside as a marker compound and not only genistein. To find out whether sophoricoside is a native constituent of *G. biloba* leaves, all 33 ginkgo samples described above were analyzed with the corresponding method.

**Figure 4.**
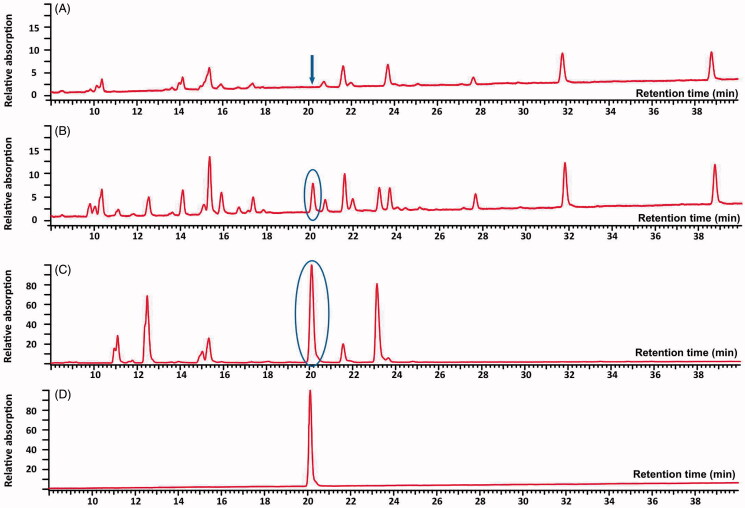
UV-detected chromatograms of *G. biloba* leaf extract (A), extract of *G. biloba* leaves with 2% adulteration of *S. japonica* fruits (B), *S. japonica* fruit extract (C) and sophoricoside (D) at 254 nm.

The UV-detected chromatograms at 254 nm of *G. biloba* extract, an extract of *G. biloba* leaves with an intentional 2% adulteration of *S. japonica* fruits, *S. japonica* fruit extract and the reference compound sophoricoside were compared (Figure 4). Sophoricoside with a retention time of 20.12 min could not be detected in any of the 33 ginkgo extracts, whereas it could be detected in the sample intentionally adulterated with *S. japonica* fruit extract.

## Discussion

When reproducing the method described by López-Gutiérrez et al. (2016), the coelution of genistein and apigenin became evident by using LC-HRMS-MS. A trace peak with the same retention time and monoisotopic mass as the genistein reference substance, but with different MS fragmentation patterns was observed in the chromatogram of ginkgo extracts (Table 1 and Figure 2). This peak could eventually be identified as apigenin by its fragmentation pattern and by comparison with a reference compound. Moreover, the UV spectrum of apigenin is different from genistein, with maxima at 336 nm and 261 nm, respectively (Mabry et al. 1970). Thus, apigenin was identified as a genuine constituent of *G. biloba* leaves, while genistein could not be detected in any of the tested samples. This confirms findings by Avula et al. (2015), that *G. biloba* leaf and its extracts do not contain genistein and that it is a marker of adulteration. Also, Wohlmuth et al. (2014) stated that the presence of genistein in ginkgo leaf extract products is considered as evidence of adulteration. However, so far, it was not clear why other authors described genistein as a native constituent in ginkgo leaf extracts (Yao et al. 2017), why some even claimed to have isolated and identified genistein from ginkgo leaf extract (Wang et al. 2007). The lack of data such as the fragmentation patterns or considerations of coeluting isomers could be the reason for the misidentification of apigenin as genistein. Also, for chemotaxonomic reasons, it is quite unlikely that genistein is contained in *G. biloba*, because isoflavonoids are typical for Fabaceae species. It is also possible that adulterated material has been used in the described studies. This clearly shows the importance of documentation of the botanical authenticity of the investigated raw materials.

Sophoricoside, the glucoside of genistein can be used as another marker compound to detect adulterations of *G. biloba* leaves with *S. japonica* fruits without prior hydrolysis of the extracts, making it a suitable method for screening a high number of samples. Using the suggested HPLC method for the analysis of sophoricoside, we were able to detect a ≥2% adulteration of *G. biloba* leaves with *S. japonica* fruit in the investigated extract as shown in Figure 4.

## Conclusions

Our results have shown that genistein and sophoricoside, which are constituents of *S. japonica* fruits, could not be detected in *G. biloba* leaves using HPLC with UV and HRMS detection. Thus, both genistein and sophoricoside are suitable marker compounds for detecting adulterations of *G. biloba* leaves with *S. japonica* fruits on an extract level, and HPLC analysis can be an important tool for monitoring the authenticity and purity of *G. biloba* leaf extracts.
